# Endogenous and Exogenous Regulatory Signaling in the Secretory Pathway: Role of Golgi Signaling Molecules in Cancer

**DOI:** 10.3389/fcell.2022.833663

**Published:** 2022-03-23

**Authors:** Simona Del Giudice, Valentina De Luca, Seyedehnegar Parizadeh, Domenico Russo, Alberto Luini, Rosaria Di Martino

**Affiliations:** Istituto per l'Endocrinologia e l'Oncologia Sperimentale “Gaetano Salvatore” (IEOS) – Sede Secondaria, Napoli, Italy

**Keywords:** Golgi, secretory pathway, secretion, protein transport, cancer, control system, signaling

## Abstract

The biosynthetic transport route that constitutes the secretory pathway plays a fundamental role in the cell, providing to the synthesis and transport of around one third of human proteins and most lipids. Signaling molecules within autoregulatory circuits on the intracellular membranes of the secretory pathway regulate these processes, especially at the level of the Golgi complex. Indeed, cancer cells can hijack several of these signaling molecules, and therefore also the underlying regulated processes, to bolster their growth or gain more aggressive phenotypes. Here, we review the most important autoregulatory circuits acting on the Golgi, emphasizing the role of specific signaling molecules in cancer. In fact, we propose to draw awareness to highlight the Golgi-localized regulatory systems as potential targets in cancer therapy.

## Introduction

Making cancer curable is one of the compelling demands of the 21st century. Our knowledge of the multifactoriality and diversity of cancer has exploded in the last 20 years, accentuating how these conditions are present not solely among different tumors but likewise among patients with the same type of tumor. They serve as both a difficulty and an opportunity, as current treatment strategies are progressing towards precision medicine. Indeed, the research and identification of new prognostic factors and therapeutic targets has turned into a foremost priority for both the academic and pharmaceutical fields. The great heterogeneity of the aspects that characterize cancer prompted the initial definition of the hallmarks of cancer to appear about 20 years ago, producing the first systematic definition of the distinctions between tumor cells and normal cells and establishing the foundations for more inquiry in the field (Hanahan and Weinberg, 2000). From that moment on, a widening number of factors have further enlarged the picture of cancerous cells, depicting a scenario that appears complex and not devoid of anomalies as published in several excellent reviews (Hanahan and Weinberg, 2011). A significant concept that has developed from the characterization of the hallmarks of cancer is that they are interconnected through regulatory circuits. Moreover, many cancer phenotypes rely on the exploitation of basic cellular processes, such as the complex structure of intracellular organelles that generates the secretory pathway.

Indeed, a growing number of discoveries have highlighted the role of the secretory pathway as a central node for tumor initiation, growth, and metastasis (Lhomond et al., 2015; Robinson et al., 2019). The functions of the secretory pathway, such as secretion, glycosylation and transport of proteins, are not only essential but also highly changed in malignant cells. In the last decade, thanks to the identification of different auto-regulatory signaling pathways within the secretory apparatus, the vision of this fundamental cellular system has gone from static and constitutive to dynamic and regulated (Cancino and Luini, 2013). It is therefore not a great surprise that signaling proteins in the secretion pathway, and in particular on the Golgi complex, play a crucial role in the maintenance and progression of cancer. With this review, we aim to focus attention on the fundamental role that the Golgi complex has at the crossroads of multiple cellular signals and functions, and how their alterations can influence the establishment and evolution of different cancers. We focused mainly on Golgi-localized signaling molecules that are important regulators of Golgi functions such as protein transport, with some exceptions justified by the cancer relevance of important Golgi-associated proteins. Indeed, this background may be of great interest in the identification of novel drug targets, as well as early biomarkers, among Golgi-localized proteins.

## Golgi Structure and Functions

The Golgi complex (GC), identified and named in 1898 by Camillo Golgi, is an intracellular membrane-bound organelle with an essential role in post-translational modification and trafficking for proteins and lipids. Golgi functions, mainly protein transport, sorting and glycosylation, emerge from its structure, based on the arrangement of several discrete cisternae that contain glycosylation enzymes and other Golgi resident components to process cargo proteins and lipids (Munro, 1998; Nakano and Luini, 2010; Huang and Wang, 2017). Initially, it was proposed that the Golgi cisternae in mammalian cells were extensively interconnected, and that the GC comprised a single set of stacked cisternae (Farquhar and Palade, 1981). Today, we know the GC is organized as a Golgi ribbon comprising multiple Golgi stacks, which are often laterally linked by tubular structures (Huang and Wang, 2017). Each of these Golgi stacks is formed by three to six tightly aligned flattened cisternae named *cis*-, *medial*-, and *trans*-cisterna based on their position regarding the ER. As mentioned before, protein and lipid transport is one of the major role of the GC. Indeed, membrane fluxes coming to the GC from the ER are dynamically compensated by intense COPI-mediated retrograde transport, and formation of Post-Golgi carriers at the level of the TGN. Retrograde transport by COPI vesicle is also used to recycle Golgi enzymes and preserve their localization within specific Golgi cisternae, as described by the cisterna maturation model. Indeed, the acquisition of a correct glycosylation requires that Golgi enzymes act with ordered-manner from *cis*-to *trans*-Golgi and thus their localization is important for the correct post-translational modification of proteins and lipids. Given this brief scenario, it is not surprising that alterations in Golgi structure and function have been found in many diseases, especially in the cancer field. Indeed, glycosylation profile alterations are common phenotypes in cancer (Mereiter et al., 2019; Costa et al., 2020) but their understanding so far has been limited to use them as tumor biomarker (Engelen et al., 2000), despite several evidences support their direct role in promoting carcinogenesis (Nguyen et al., 2017). The best known example involves the aberrant glycosylation profile that is found on several membrane receptors, like the EGFR, with the consequence of hyper-activation of the downstream signaling (Freitas et al., 2019). The roles of glycosylation in cancer have been already excellently reviewed (Mereiter et al., 2019; Costa et al., 2020). Thus, here we focus mainly on the role of signaling proteins on the Golgi in different cancer associated phenotypes, with a specific inset on the characteristic of metastatic cancers such the migration and invasion.

## Signaling on the Golgi: Control Systems for Protein Transport

In the last decade, the research in the membrane transport field has led to the discovery of several regulatory systems to control specific steps of protein transport in mammalian cells. Briefly, proteins start their journey from the ER, to reach the Golgi complex, and are subsequently sorted to different cellular compartments thanks to specific sorting signals (De Matteis and Luini, 2008). Specifically, the above mentioned steps in protein transport are the ones mostly endowed with regulatory signaling pathways. Importantly, most signals are organized according to the principles of the control system theory.

**FIGURE 1:**
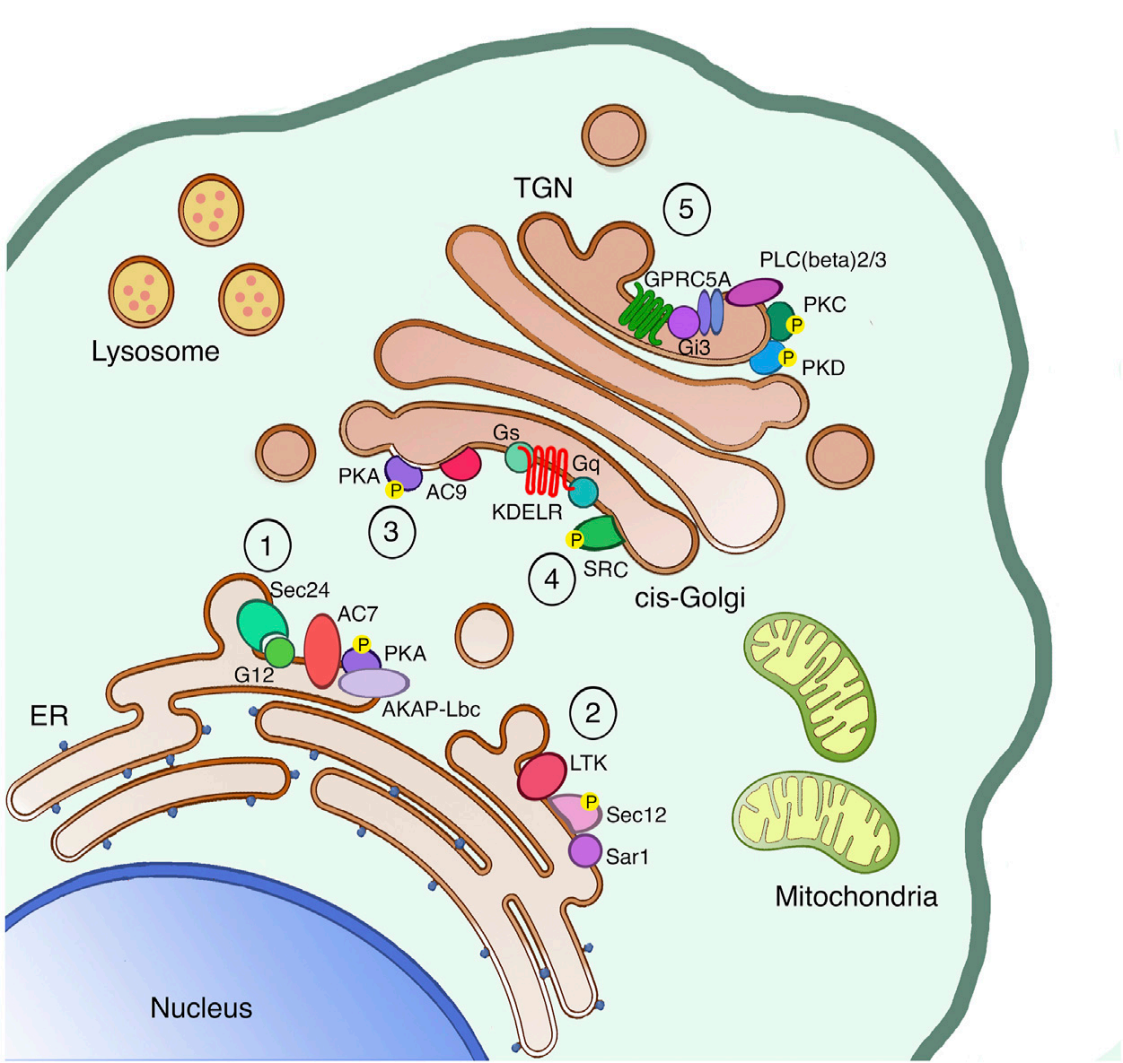
The secretory pathway is a signaling platform. The secretory pathway hosts several signaling machineries, whose function as autoregulatory systems has been characterized in detail. The ER localized AREX complex (1) and LTK-dependent system (2) are involved in regulating ER export of different proteins. Once proteins leave the ER, also ER resident chaperones reach the cis-Golgi, where they bind and activate the KDELR. Indeed, the KDELR activation stimulates both the Gs-PKA signaling (3) to regulate retrograde traffic and the Gq-SRC pathway (4) to promote the anterograde transport through the Golgi. Finally, proteins reach the TGN to be sorted and reach the final destination either inside or outside the cell. Specifically, basolateral proteins stimulate the GPRC5A receptor to activate PKD in order to exit the TGN (5).

A control system is a set of components connected or interacting with each other, which allows the variable output to be maintained stable in relation to internal or external perturbations. Different components allow the behaving of the control system, among which the crucial ones are the sensor, the controller and the effector. The major difference between a homeostatic control system and a general signaling response is that homeostatic control systems react to perturbation to reestablish the original status of the cell. Conversely, the signaling response to an extracellular perturbation stimulates a signaling cascade that alters the initial cellular status. Homeostatic control systems have a fundamental role in physiology, since they govern the capability of the body to maintain constant parameters such as blood pressure, oxygen levels, glucose concentration, and so on. Control systems have been found also at cellular levels with relevant functions in the cell-cycle regulation, energy metabolism and membrane transport. Below, we illustrate the principal control systems uncovered so far in the secretory pathway and summarised in Figure 1. Then in the following sections we define in details the key evidences supporting the role in cancer of the specific Golgi-localized signaling molecules.

### ER-Localized Control Systems

The control mechanism already starts at the ER level, where we find a first auto-regulation system capable of managing the various fluctuations in the amount of unfolded proteins in the ER lumen, and thus restoring their balance. This system is called the unfolded protein response (UPR) and it is activated by the accumulation of unfolded or mis-folded proteins in the ER lumen. The UPR helps the ER to eliminate these toxic proteins through acute and transcriptional responses, excellently reviewed elsewhere (Gardner et al., 2013). Another ER-localized regulatory system is the one responding to the Leucocyte tyrosine kinase (LTK). LTK phosphorylates Sec12, the integral ER protein responsible for Sar1 activation on ER-exit sites (ERES), thus LTK activity regulates ER-export and ERES number (Centonze et al., 2019). Recently, a new ER auto-regulation system called AREX (auto-regulation of ER export) has been identified, and it is capable of taking over the fluxes of folded cargo leaving the ER and arriving at the Golgi complex. This system dwells in the sequential recruitment and activation specific ER-localized signaling components (such as Sec24, G alpha 12, the Adenylate cyclase 7, the phosphodiesterase 3B and AKAP-lbc). This signaling complex favors the packaging of the cargo in COPII coated vesicles, and the translocation of these carriers towards the *cis*-side of the GC (Subramanian et al., 2019) where they stimulate other regulatory control systems.

### *cis*-Golgi Localized Auto-Regulatory Control Systems

At the *cis*-Golgi, we identify two regulatory systems for the anterograde and the retrograde transport. These control systems can restore the equilibrium and homeostasis of the Golgi complex and other compartments in the event of system perturbations. Moreover, these two mechanisms are both regulated by specific receptors structurally belonging to the sweet transporter family, named KDEL receptor (KDELR). The KDELR is a group of three proteins (KDELR1/2/3), characterized by the presence of a seven-transmembrane domain, and localized in the ER and *cis*-Golgi. The KDEL receptor is stimulated by the KDEL sequence of ER chaperones, promoting the recycling of chaperones between the ER and *cis*-Golgi compartments. Once activated, the KDELR also increases the anterograde transport from the *cis*-to the *tran*s-Golgi by stimulating the Gq alpha subunit of the heterotrimeric G-proteins localized on the Golgi. Gq in turn activates the protein kinase SRC and its downstream signaling cascade (Pulvirenti et al., 2008; Giannotta et al., 2012). The KDELR also switches on the alpha G-protein Gs, which controls the *cis*-Golgi to ER retrograde transport through the activation of the catalytic subunit of the protein kinase A (PKA) on the Golgi (Cancino et al., 2014). Active PKA then phosphorylates specific substrates that, on one hand, support the return of the KDELR/chaperone complex to the ER in COPI coated vesicles, and on the other promote the transcription of distinct genes (Cancino et al., 2014). More recently, two new KDELR signaling mechanisms have been identified. In the first, the KDELR regulates the activation of the G protein alpha 0 to improve the elongation and stability of protrusions on plasma membrane (Solis et al., 2017). In the second, the KDELR1 induces the relocation of the lysosomes from the cell periphery to the Golgi area, and the formation of auto-phagosomes, thanks to a Gs/PKA dependent phosphorylation of the protein dynein-LRB1 (Tapia et al., 2019).

### TGN Localized Auto-Regulatory Control Systems

Moving forward in the secretory pathway, at the Trans-Golgi Network (TGN) cargo proteins are sorted to specific membranes such endo-lysosomes or the plasma membrane, or secreted in the extracellular space. For this reason, the TGN trafficking and sorting machinery need to recognize specific signals, likewise known as sorting motifs, present on the vast majority of cargo proteins. In polarized epithelial cells, post-Golgi carriers containing plasma membrane proteins can be targeted to the apical or basolateral surfaces (Bard and Malhotra, 2006). For the sorting of apical proteins, individual post-translational modifications such as glycans are important, while the sorting of basolateral proteins involves the synergic activation of signaling and transport machinery where the serine/threonine protein kinase D (PKD) shows a key role. Once the basolateral cargoes arrive at the TGN, they activate the βγ subunits of heterotrimeric G proteins to stimulate a downstream signaling cascade that directly targets the PLCβ 2/3 phospholipases. PLCβ 2/3 enzymes hydrolyze the TGN pool of phosphatidylinositol 4,5-bis phosphate (PIP2), producing the second messengers DAG and inositoltryphosphate (IP3). The DAG has the double function of activating the TGN-associated protein kinase C η (PKC η) and to recruit PKD, thanks to its cysteine-rich regions, on TGN membranes. PKCη then phosphorylates and activates PKD (Bard and Malhotra, 2006). PKD can phosphorylate different substrates on the TGN (Fugmann et al., 2007; Nhek et al., 2010), including the phosphoinositide kinase PI4KIIIB (Hausser et al., 2005).

Remarkably, PKD and PI4KIIIB are also part of a TGN localized complex that includes the protein BARS (brefeldin A ADP-ribosylation substrate), the 14-3-3γ protein and the p21-activated kinase (PAK) required for the fission of basolaterally targeted post-Golgi carriers (Valente et al., 2012; Pagliuso et al., 2016). PAK1 and PAK4 are localized on TGN membranes and are required for the formation of post-Golgi carriers containing basolateral cargoes, and BARS is one of the PAKs substrates on the TGN (Valente et al., 2012). More recently, PKD action on regulating basolateral proteins transport has further expanded, thanks to the identification of PARP12 as a novel TGN localized substrate (Grimaldi et al., 2022). Indeed, PARP12 catalyzes the specific ADP-rybosilation of Golgin97 during TGN export of important basolateral proteins, such as E-cadherin. PKD-dependent PARP12 phosphorylation has a role in regulating this signaling network (Grimaldi et al., 2022). Activated PKD is involved in several pathways on the Golgi complex, not only for the export of basolateral proteins (Malhotra and Campelo, 2011) but also for the entry into mitosis by driving Golgi fragmentation in the G2 phase of the cell cycle (Kienzle et al., 2013). Exogenous signals and stimuli can lead also to an activation of PKD signaling on the Golgi. One possibility, for example, is the production of second messengers that allow the signaling pathway originated in the plasma membrane to reach other cellular districts. Alternatively, the extracellular signaling can reach other cellular organelles thanks to the translocation of signaling molecules to the target compartment. For example, upon GPCR stimulation on the plasma membrane, the protein GEF-H1 translocates from the microtubules and reaches the TGN. This allows GEF-H1 to start the signaling cascade that activates PKD via RhoA to promote transport to the focal adhesions (Eisler et al., 2018). Indeed, focal adhesions are particularly important for the secretion of MMPs and extracellular matrix remodeling, and thus the molecules involved in this signaling have a specific relevance in the context of tumor invasion (Heck et al., 2012).

Recently, a new auto-regulatory mechanism has coupled the arrival of basolateral cargo proteins to the activation of signaling molecules acting upstream PKD signaling, thanks to the identification of the orphan receptor GPRC5A. GPRC5A is localized on TGN and plasma membrane, it has a seven transmembrane domain like all GPRC proteins, and it is associated with heterotrimeric G proteins. GPRC5A stimulates the activity of βγ-PLCβ-PKCη-PKD and of the βγ-PAK signaling cascades upon the formation of a physical complex with the basolateral cargo proteins. This mechanism is called AutoRegulation of TGN export (ARTG) and appears to have a key role in the maintenance of apico-basal polarity and secretion in epithelial cells (Di Martino et al., 2020). The role of these auto-regulatory systems is to ensure the homeostasis of the system and the optimal performance of the various transport stations. This system also avoids imbalances in the flows of membranes or accumulation of proteins in erroneous cellular compartments with potentially toxic effects not only at the cellular level but also of the organism.

## Golgi Signaling Molecules and Cancer

As mentioned in the previous section, the Golgi complex hosts several signaling molecules, especially G proteins and kinases. These molecules are components of signal transduction pathways operating at the Golgi to control its dynamic organization and function (Sallese et al., 2006). Golgi-localized signaling proteins also take part in regulating general cellular processes, such as proliferation, differentiation and motility (Bard et al., 2003; Mavillard et al., 2010).

**FIGURE 2:**
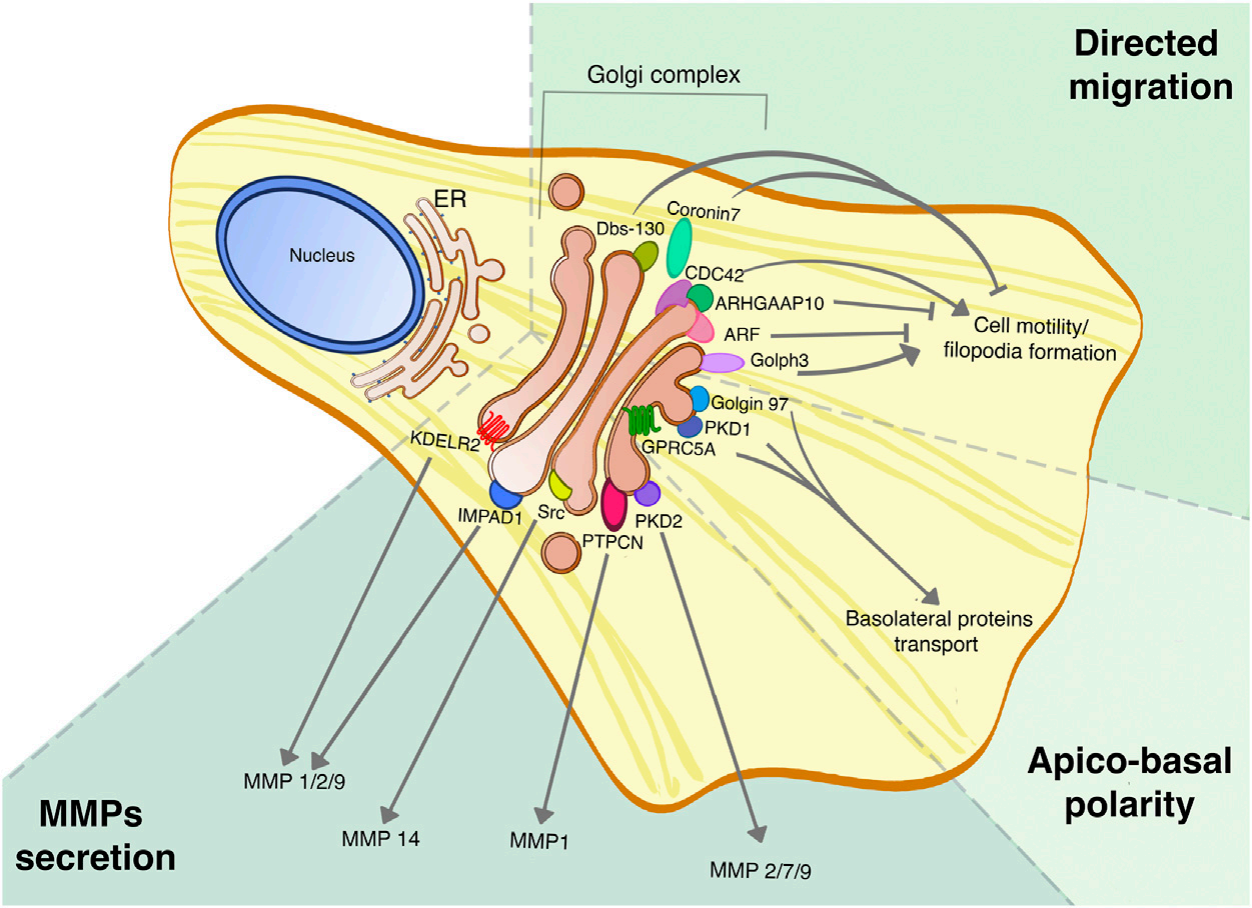
Golgi proteins roles in tumor dissemination and invasion. Golgi proteins are involved in multiple processes linked to tumor aggressiveness, such as the secretion of MMPs, cell migration and regulation of apico-basal polarity, and their roles have been assessed in different tumors. This picture summarizes the different Golgi signaling proteins involved in regulating specific MMPs secretion, the Golgi-localized proteins that are part of the CDC-42 complex for directed cell migration, and finally the PKD signaling pathway that promotes the maintenance of apico-basal polarity.

Because of their widespread involvement in regulating fundamental cellular functions, many Golgi-based signaling molecules show a correlation with cancer, and in particular with phenotypes that are associated with more aggressive tumors. Clinically, tumor aggressiveness is defined as rapid formation, growth, or spread, usually accompanied by a negative outcome for the patient. In this scenario, the spreading of the primary tumor is associated with the dissemination of circulating cells and invasion of nearby or distant sites to form metastases, that actually are the primary cause of death for cancer patients (Steeg, 2006). Cancer metastasis formation is a complex mechanism, characterized by a cascade of events that can be summarized as loss of cell-cell adhesion to detach from the primary tumor mass, secretion of matrix metallo-proteases (MMPs) to degrade both the basal membrane and the extracellular matrix, and also enhanced cell motility (Valastyan and Weinberg, 2011; Fares et al., 2020). The Golgi complex is strongly involved in several of these processes, such as secretion of MMPs, regulation of cell motility and maintenance of correct apico-basal polarity, as highlighed in Figure 2 (Millarte and Farhan 2012; Goldenring, 2013; Olayioye et al., 2019).

MMPs are a family of 23 proteins, divided into four classes based on their substrate specificity, whose extracellular secretion occurs in a Golgi-dependent fashion (Klein and Bischoff, 2011b). The constant interest in regulating MMPs secretion is because of their essential role in the remodeling of extracellular matrix, especially for their pivotal action in sustaining cancer cell motility and invasion (Gialeli et al., 2011). Despite this, the research and design of effective MMPs inhibitors has not been given the expected results so far (Fields, 2019), hence an alternative approach to target MMPs could derive from targeting their mechanism of secretion. Although the specific mechanisms that promote the transport and secretion of soluble proteins are not completely understood, several Golgi proteins are involved in MMPs trafficking and secretion.

The acquisition of a highly motile phenotype is a characteristic of metastatic cells, but in a more physiological context, it also takes part in wound healing and tissue development. Cell migration from the primary tumor during the EMT can happen both as a single cell, giving rise to circulating cancer cells, or as collective migration (Lintz et al., 2017). The role of the Golgi complex in cell migration is very well understood, has been highlighted in several reviews. Indeed, the position of the Golgi regarding the nucleus is important for the definition of the front-rear polarity in migrating cells, but also several Golgi proteins play a crucial role either promoting or inhibiting cell migration.

As said in the previous paragraph, the principal functions of the Golgi are protein transport and Glycosylation. It is thus not surprising that the Golgi complex plays a pivotal role in the establishment and maintenance of apico-basal polarity, since most of it relies on the correct sorting at the level of the TGN. The TGN is the major sorting station of the cell, and here a synergy of cytosolic factors, signaling molecules and protein sorting motifs, coordinates the success of protein sorting to the correct destination. This has been studied in depth, especially for basolateral transmembrane proteins (Weisz and Rodriguez-Boulan, 2009; Guo et al., 2014; Di Martino et al., 2019). Hence, here we review some of the best-known examples of the roles of Golgi proteins, especially signaling molecules, in cancer.

### The KDEL Receptors

The KDEL receptors represent an important class of proteins (Capitani and Sallese, 2009) whose complete functions are still poorly understood (Mao et al., 2020). So far, the KDEL receptor is associated with regulating membrane protein traffic (Giannotta et al., 2012), cell proliferation and immune responses (Kamimura et al., 2015), ER stress, and autophagy (Yamamoto et al., 2003; Wang et al., 2011). Recent studies revealed that KDELR expression levels are increased in tumors and it is involved in tumor progression and metastasis formation (Bajaj et al., 2020; Liao et al., 2019; Marie et al., 2020).

Indeed, KDELR2 overexpression promotes the metastatic phenotype in non-small cell lung cancer (NSCLC) by increasing MMP1, MMP2 and MMP9 secretion and thus cell invasive ability (Bajaj et al., 2020). The proposed model suggests that increasing KDELR levels speeds up the retrograde transport of KDEL-containing chaperones, which bind to MMPs in the ER and transport them to the Golgi complex before being recycled by the KDELR. The KDELR2 is highly expressed in glioblastoma (GBM) tissues and negatively correlated with patients’ survival (Liao et al., 2019). Indeed, KDELR2 protein levels strongly correlate with HIF1α, a potent activator of proliferation, invasion, and angiogenesis in the GBM context. HIF1α directly binds the HRE region within KDELR2 promoter, and enhances the KDELR2 transcription (Liao et al., 2019). Besides this, the KDELR2 overexpression in GBM cells significantly increases mTOR phosphorylation, suggesting that KDELR2 could activate the mTORC1 pathway, which is correlated with cell proliferation and tumor aggressiveness (Ghosh and Kapur 2017). The KDELR2 has been identified also as one driver of invasion and metastasis in lung cancer together with IMPAD1, a Golgi resident phosphatase (Bajaj et al., 2020). IMPAD1 has a fundamental role in the sulfation of glycosaminoglycans, thus its loss of function mutation implies severe bones and skeletal defects in human patients (Vissers et al., 2011). IMPAD1 overexpression increases MMP1, MMP2 and MMP9 secretion in NSCLC, although the underlying molecular mechanisms are not completely clear (Bajaj et al., 2020).

### Arf Family

The Arfs proteins are part of a larger family of small GTP-binding proteins, that also includes the Arf-like (Arl) proteins. These proteins are involved in a variety of cellular processes such as membrane trafficking, targeting of proteins to cilia, microtubule regulation, and lysosome function. There are three classes of mammalian Arf proteins: Class I (Arfs1-3), Class II (Arfs 4,5), and Class III (Arf6). Class I Arfs are highly conserved in evolution and play an essential role in maintaining the structure and function of the Golgi complex (Gillingham and Munro, 2007; Manolea et al., 2010; Donaldson and Jackson, 2011; Jackson, 2018).

At the Golgi complex, the Arf proteins promote membrane recruitment of many cytoplasmic coat proteins, allowing the selection of membrane proteins for transport. Moreover, they stimulate the activity of enzymes that regulate Golgi lipid composition, and assemble a cytoskeletal scaffold on the Golgi (Jackson, 2018). Based on their involvement in different cellular processes, Arf family members have also emerged as potential regulators of cancer progression. In fact, their expression is altered in a variety of cancers, including breast cancer, colon cancer, lung cancer, ovarian cancer, and hepatocellular carcinoma. For instance, Arf1 regulates cell-cell adhesion by inhibiting the formation of Adherens junction (AJs) in an E-cadherin-dependent manner (Schlienger et al., 2016). Additionally, Arf1 regulates the formation of Focal Adhesions (FAs) in invasive breast cancer cells through the phosphorylation of FAK (Schlienger et al., 2015). Also Arf6 is involved in both AJ assembly and disassembly. Indeed, Arf6 regulates the internalization and transport of E cadherin between the basolateral plasma membrane and the early endosomes, facilitating the degradation of AJ (D’Souza-Schorey and Chavrier, 2006). Furthermore, Arf proteins have also been described as key players in regulating actin remodeling. Both Arf1 and Arf6 induce changes in the actin cytoskeleton by regulating the trafficking of Rac1, a small GTPase, which is known to regulate the migration of cancer cells (Lewis-Saravalli et al., 2013).

### Golgi Protein Kinases

As described above, several protein kinases play an important role in transport and signaling at the Golgi. It is well known that protein kinases regulate a myriad of cellular processes, such as cell division, metabolism, survival and apoptosis, hence their deregulation is strongly associated with cancer initiation and tumor progression.

#### Src Family Kinases

The Src family kinases (SFKs) are a family of non-receptor tyrosine kinases responsible for signal transduction during many cellular events, such as differentiation, adhesion, and migration. This family is composed of nine members, whose distinguish feature is a modular structure containing the Src homology domains 2 and 3 (SH2 and SH3), which are involved in protein-protein interactions (Espada and Martin-Perez, 2017; Shah et al., 2018; Mayoral-Varo et al., 2021). Altered Src activity is associated with enhanced tumor progression, and several mechanisms have been proposed to explain it. Many tumor types, such as prostate cancer, have high expression or activity of several growth factor receptors, like the epidermal growth factor receptor (EGFR), platelet-derived growth factor receptor (PDGFR), and vascular endothelial growth factor receptor, which perform their functions by activating Src (Blume-Jensen and Hunter, 2001; Pietras et al., 2003; Ferrara, 2004; Harari, 2004). In this context, it has been showed that KDELR controls the ability of cancer cells to degrade the ECM through the Src pathway. Indeed, the KDELR induces Src activation and leads to phosphorylation of the Src substrates Cortactin and ASAP1, which are required for ECM degradation (Ruggiero et al., 2015). The overexpression of Src kinases results in Golgi fragmentation in pancreatic cancer cells and secretion of angiogenic growth factors (Kim et al., 2009; Weller et al., 2010).

Moreover, Src kinase regulates the correct localization of several members of the N-acetylgalactosaminyltransferase (GALNTs) family of Golgi enzymes (Gill et al., 2010). Hyper-activation of Src causes ER-localization of GALNTs, thus increasing their activity (Gill et al., 2010). Recently, Src has been shown to increase the rate of GALNT retrograde traffic through the phosphorylation of GBF1, thus regulating the GBF1-Arf1 interaction (Chia et al., 2021). GBF1 acts as GTP exchange factor (GEF) in the early *cis*-Golgi and ER-Golgi intermediate compartments (ERGIC) to regulate retrograde traffic from the Golgi to the ER (Kawamoto et al., 2002; Zhao et al., 2002; Zhao et al., 2006). In normal cells, the interaction of GBF1 with Arf is limited and retrograde traffic rate is moderate. Conversely, in tumor cells GBF1 is phosphorylated upon Src activation, which results in increased affinity for Arf-GDP on Golgi membranes (Chia et al., 2021). These reactions lead to the formation of tubules containing GALNTs and the enzymes’ relocation to the ER, wherein they glycosylate resident and neo-synthesized substrates. One of the biological effects of GALNTs Activation, named GALA pathway, is the MMP14 glycosylation operated by the ER-relocalized GALNT1 enzyme (Nguyen et al., 2017). Indeed, glycosylated MMP14 leads to increased extracellular matrix degradation and promotes liver tumor metastasis (Nguyen et al., 2017).

#### Protein Kinase A

PKA is one of the most important member of the large serine-threonine protein kinase superfamily, and is involved in the control of a variety of cAMP-dependent cellular processes. It has been reported that PKA is implicated in the initiation and progression of many tumors (Naviglio et al., 2009), also through the role of its phosphorylated substrates. Indeed, the cAMP response element binding protein (CREB), one of the most important target proteins of PKA, is a transcription factor that regulates the expression of several oncogenes, including c-Jun and cyclin D1 (Steven et al., 2020). Notably, cAMP–PKA–CREB signaling has both tumor-suppressive and tumor-promoting effects, depending on the tumor types and context (Zhang et al., 2020).

Another substrate of PKA is the CDC42 interacting protein 4 (CIP4), a CDC42 effector that interacts with A-kinase (PKA) anchoring protein 350 (AKAP350) on the Golgi apparatus (Aspenström, 1997; Fricke et al., 2009), and it coordinates membrane deformation and actin polymerization for the acquisition of a more motile phenotype. Recently, it was shown that CIP4 phosphorylation by PKA depends on AKAP350 expression (Tonucci et al., 2019). The distinct lines of evidence suggesting a functional interaction between CIP4 and AKAP350 during the acquisition of invasive properties (Tonucci et al., 2015; Hu et al., 2016) suggested that PKA regulates CIP4 function during the development of invasive properties in cancer cells. PKA can also inactivate the calmodulin-dependent protein kinase kinase-2 (CAMKK2) (Langendorf et al., 2020). The inhibition of CAMKK2 protects against prostate cancer, hepatocellular carcinoma (HCC) and metabolic disorders induced by a high-fat diet (Lin and Wu, 2015). Moreover, it is known that PKA can reprogram lipid metabolism by inhibiting salt-inducible kinases (SIK1-3), and then promote pancreatic tumorigenesis (Patra et al., 2018).

#### Protein Kinase D

Protein kinase D is a family of three members (namely PKD1, PKD2 and PKD3), which localizes also on TGN membranes and regulates protein secretion. Given PKD involvement in many cellular functions, consistent evidence links PKD to a variety of signaling pathways involved in tumor development and cancer progression. Indeed, different tumors are characterized by aberrant expression of PKDs. For example, in highly invasive breast cancer, losing PKD1 promotes invasion and metastasis, while upregulated PKD2 and PKD3 positively regulate proliferation, chemo-resistance and metastasis (Durand et al., 2015).

Indeed, PKD2 regulates the constitutive secretion of MMP2, MMP7 and MMP9, forming a protein complex that involves the small GTPases ARF1 and ARL1, and Arfaptin2. This complex is distinct from the complex needed for basolateral transmembrane proteins transport (Eiseler et al., 2016). Moreover, many mitogenic GPCR agonists that mediate their response through Gq, G12 and Gi can activate PKDs. In Swiss 3 T3 cells, PKD overexpression can potentiate DNA synthesis in response to bombesin and vasopressin, leading to increased cell proliferation (Roy et al., 2017). PKD is also implicated in regulating cell survival and apoptosis through modulation of the NF-κB and c-Jun N-terminal kinase (JNK) pathways (Roy et al., 2017).

As mentioned above, PKD activation regulates PARP12 dependent ADP-rybosilation of Golgin 97 on TGN membranes (Grimaldi et al., 2022), and both PARP12 and Golgin97 have clear roles in cancer. Indeed, low Golgin97 expression has been correlated with increased invasiveness and poor survival in breast cancer patients, and Golgin97 depletion in breast cancer cell lines leads to hyper-activation of NF-kB and reduced IκBα, thus promoting migration and invasion (Hsu et al., 2018). Similarly, PARP12 depletion has been associated with increased migration and metastasis in hepatocellular carcinoma (Shao et al., 2018), and to resistance to genotoxic stress in breast cancer cell lines (Gaston et al., 2016).

#### p21-Activated Kinases

The family of p21-activated kinases (PAKs) comprises six members in the human genome and they have clear roles in regulating cell motility and polarity (Zhao and Manser, 2012). PAKs are cytosolic proteins but they can be recruited to different membranes, including the Golgi complex as with PAK4 (Larocca et al., 2004) Indeed, PAKs are targets of the small GTPases cdc42 and Rho, and many PAKs substrates are cytoskeletal regulator proteins, such as LIM kinase, myosin light-chain kinase (MLCK) and Cortactin, or also regulators of membrane trafficking like BARS (Barnes et al., 2003; Valente et al., 2012; Kumar et al., 2017). PAK proteins can also activate the mitogenic signalling by phosphorylation of Raf1 (Sun et al., 2000), hence it is not a surprise that PAKs have been involved in several aspects of tumorigenesis (Kumar and Li, 2016; Sun et al., 2000). Indeed, PAK1 pathway is one of the most hyper-activated signaling in human cancer, but also PAK2 and PAK5 are frequently over-expressed in several types of tumors (Kumar and Li, 2016).

### Cdc42

The small GTPase Cdc42, whose localization is both on the Golgi and on the plasma membrane, is well known for its role in cell migration (Farhan and Hsu, 2016). Loss or inhibition of Cdc42 have a powerful effect on Golgi organization and reduce cell motility (Friesland et al., 2013; Xiao et al., 2018), while its overexpression has been linked to more metastatic phenotypes, even if with some exceptions (Stengel and Zheng, 2011). On the other hand, some Golgi localized proteins negatively regulate Cdc42 activity. Their loss induces increased cell migration through the protrusion of actin-rich filopodia, hence they have a tumor suppression function in several cancer types (Bhattacharya et al., 2016; Teng et al., 2017). The specific Golgi-localized Cdc42 Guanine activating proteins (GAPs) ARHGAP10 and Dbs-130 give the most known cases, negatively regulating Cdc42 by increasing its GTPase activity (Dubois et al., 2005; Fitzpatrick et al., 2014). Also non-GAP proteins might have similar functions. This is the case of the actin-binding protein Coronin 7, that binds Cdc42 on the Golgi and regulates the function of the Cdc42 effector n-WASP (Bhattacharya et al., 2016).

### GPRC5A

The family of G-protein-Coupled Receptors (GPCRs) is the largest family of transmembrane proteins, comprising over 800 GPCRs identified so far (Katritch et al., 2013). Amongst them, the orphan receptor GPRC5A has the double feature of being strongly involved in cancer (Acquafreda et al., 2009; Zhou and Rigoutsos, 2014; Jiang et al., 2018) and of being localized on Golgi associated vesicles and plasma membrane (Zhou and Rigoutsos, 2014; Hirabayashi and Kim, 2020; Di Martino et al., 2020 BioRxiv). GPRC5A can have the role of tumor suppressor or of oncogene depending on the different cancer types (Acquafreda et al., 2009; Zhou and Rigoutsos, 2014), adding more complexity to the unraveling of its cellular signaling and molecular mechanisms. GPRC5A is one of the four members of the family of orphan retinoic acid inducible GPCRs (Cheng and Lotan, 1998; Robbins et al., 2000), whose expression is chordate specific (Kurtenbach et al., 2011). In healthy tissues, GPRC5A is mostly found in the lungs and its cellular signaling has been linked to the regulation of EGFR activity (Song et al., 2019; Wang et al., 2016; Zhong et al., 2015; Lin et al., 2014).

More recently, our research group is highlighting GPRC5A involvement in a signaling complex that regulates PKD activation on TGN membranes to assure the efficient and optimal transport/secretion of basolateral proteins (Di Martino et al., 2020 BioRxiv). This finding opens to new roads to understand the molecular mechanisms underlying the higher aggressiveness and metastasization observed in tumors where GPRC5A expression is altered (Sawada et al., 2020; Dai et al., 2021).

Apico-basal polarity is also one of the major target of EMT hence proteins that are involved in regulating TGN sorting and export can suppress the metastatic phenotype, as with PKD1 (Durand et al., 2015). On the same line, GPRC5A has been identified as lung tumor suppressor both in mice and humans (Tao et al., 2007). Interestingly, GPRC5A-KO mice present EMT-like characteristics in the lungs upon treatment with silica (Wang et al., 2015). Our data support this evidence, since GPRC5A promotes basolateral proteins export by activating PKD signaling on TGN membranes (Di Martino et al., 2020 BioRxiv).

### GOLPH3

Golph3 is one of the most known “Golgi oncogenes,” as discussed in several excellent reviews (Rizzo et al., 2017; Kuna and Field, 2019; Sechi et al., 2020), and it is a potent modulator of multiple RTKs and mitogenic signaling. The cytosolic protein GOLPH3 localizes to the trans-Golgi network thanks to the interaction with PI4P (Dippold et al., 2009), which is enriched on TGN membranes. At gene level, GOLPH3 is targeted for amplification in several solid tumors (Scott et al., 2009) and it has been linked to increased activation of the Akt/mTOR pathway and cell proliferation (Scott et al., 2009; Zeng et al., 2012). GOLPH3 yeast homologue Vps74p regulates the localization of Golgi enzymes. The overexpression of Golph3 promotes also directed cell migration by increasing protein transport to the leading edge thanks to the interaction with the actin-binding protein Myosin XVIIIA (Xing et al., 2016). More recently, Golph3 has been found to regulate the localization and thus the activity of several Golgi glycosphingolipids also in mammalian cells, leading to enhanced production of specific growth-inducing glycosphingolipids and reprogramming the glycosphingolipid pathway to potentiate mitogenic signaling and cell proliferation (Rizzo et al., 2021).

### PITPNC1

The TGN protein Phosphatidylinositol Transfer Protein Cytoplasmic 1 (PITPNC1) promotes the transfer of phosphatidylinositol (PI) and phosphatidic acid (PA) between different membrane compartments. PITPNC1 is genetically amplified in breast cancers and over-expressed in several solid tumors, with a significant correlation with metastatic progression (Halberg et al., 2016). PITPNC1 binds the phosphatidylinositol-4-phosphate (PI4P) to keep its TGN localization, and thus to express its pro-metastatic effects. Indeed, PITPNC1 depletion or its displacement from the TNG reduce the secretion of MMP1 and of other pro-metastatic factors. At the molecular level, PITPNC1 interacts with RAB1B, and their overexpression increases PI4P levels on the TGN. Because of this increased PIP4 pool, also the known Golgi oncogene Golph3 increases its TGN localization, facilitating the incorporation of MMP1 and other soluble pro-metastatic proteins in the transport vesicles (Halberg et al., 2016).

## Conclusion

The centrality of the Golgi complex for many cellular and physiological functions has often attracted the attention of scientists to the role of this organelle in many areas of pathology, and in particular in the cancer field (Liu et al., 2021; Zhang, 2021). The role of Golgi in cancer can be analyzed starting from its fundamental functions, for example protein transport and glycosylation. Furthermore, the morphology of the Golgi itself is an important cancer-associated phenotype (Petrosyan, 2015). With regard to transport, our understanding of the links between traffic and cancer have been greatly helped by the studies conducted with synchronizable transport systems that have been used for decades for the characterization of transport machinery and of transport kinetics within the secretory pathway. Some technical limits of these approaches have been successfully overcome in the last 10 years with the adoption of the RUSH (retention upon selective hook) system (Boncompain et al., 2012) although this technique is still limited by the use of transfected proteins. Regardless, these protein synchronization approaches have helped in the identification of many signaling pathways that are triggered by traffic and generate regulatory loops that control protein transport in the secretory route. Strikingly, the molecular components of these pathways have often turned out to be oncogenes or tumor suppressor (as with Src, GOLPH3, PKD, and GPRC5A), a role that was sometimes discovered only after their characterization as transport regulators.

The transport associated signaling proteins are generally located in subcellular pools, from where they regulate specific transport functions. They form interactions and complexes that are specific for the organelles where they reside. It is therefore important to develop targeted approaches for the study of signaling complexes anchored to Golgi membranes, as this would be useful to develop tools for the study of Golgi-dependent cancer phenotypes. Several signalling molecules, such as SRC and PKD, can be recruited to both plasma membrane and intracellular membranes, especially Golgi, thus activating different signaling pathways. Cancer cells take advantage of the hyperactivation of these molecules because it amplifies the effects that sustain growth and/or invasion. This signaling amplification is achieved thanks to the activation of specific substrates across the different cell compartments. Moreover, the regulation of transport in the secretory pathway is affecting several classes of proteins, including many adhesion proteins and receptors. Indeed, the alteration in the mechanisms of transport is also altering their optimal function on the plasma membrane. For example, the mis-localization of adhesion proteins leads to defects in the formation of cell junctions, while plasma membrane receptors need to be efficiently transported for proper signaling. Hence for cancer cells is very advantageous to positively select mutations that affects signaling proteins involved in the regulation of the secretory pathway functions. More in general, our comprehension of the relationships between membrane transport and cancer would benefit from the identification of signaling networks that regulate all the phases of the secretory pathway.

The recent identification of new Golgi-localized oncogenes and tumor suppressors encourages the hypothesis that many other molecules of this kind have yet to be identified (McKinnon and Mellor, 2017; Obata et al., 2017; Jensch et al., 2018; Sun et al., 2020). Unfortunately, despite the correlation between Golgi-based signaling and the maintenance of specific tumor phenotypes, a systematic analysis in a broad spectrum of tumors is still missing. We expect that future studies may open up scenarios that would enhance our understanding of the role in cancer of Golgi-based oncogenes and tumor suppressors. It is plausible that such studies may also allow the identification and characterization of new molecular targets for tumor treatment. The therapeutic relevance of these findings would be remarkable when one considers that the molecules involved belong highly drugable protein classes, such as kinases and GPCRs, just to name a few. As has often happened in the past, this would be a case in which curiosity driven “basic” research would have paved the way for progress in the therapeutic arena.
